# Highly Sensitive Reflective-Mode Defect Detectors and Dielectric Constant Sensors Based on Open-Ended Stepped-Impedance Transmission Lines

**DOI:** 10.3390/s20216236

**Published:** 2020-10-31

**Authors:** Pau Casacuberta, Jonathan Muñoz-Enano, Paris Vélez, Lijuan Su, Marta Gil, Ferran Martín

**Affiliations:** 1 CIMITEC, Departament d’Enginyeria Electrònica, Universitat Autònoma de Barcelona, Bellaterra, 08193 Barcelona, Spain; Pau.CasacubertaO@e-campus.uab.cat (P.C.); Paris.Velez@uab.cat (P.V.); Lijuan.Su@uab.cat (L.S.); Ferran.Martin@uab.cat (F.M.); 2 Departamento Ingeniería Audiovisual y Comunicaciones, Universidad Politécnica de Madrid, 28031 Madrid, Spain; Marta.Gil.Barba@upm.es

**Keywords:** microwave sensors, stepped-impedance transmission lines, dielectric constant sensor, phase-variation sensors

## Abstract

In this paper, reflective-mode phase-variation sensors based on open-ended stepped-impedance transmission lines with optimized sensitivity for their use as defect detectors and dielectric constant sensors are reported. The sensitive part of the sensors consists of either a 90° high-impedance or a 180° low-impedance open-ended sensing line. To optimize the sensitivity, such a sensing line is cascaded to a 90° transmission line section with either low or high characteristic impedance, resulting in a stepped-impedance transmission line configuration. For validation purposes, two different sensors are designed and fabricated. One of the sensors is implemented by means of a 90° high impedance (85 Ω) open-ended sensing line cascaded to a 90° low impedance (15 Ω) transmission line section. The other sensor consists of a 180° 15-Ω open-ended sensing line cascaded to a 90° 85-Ω line. Sensitivity optimization for the measurement of dielectric constants in the vicinity of that corresponding to the *Rogers RO4003C* substrate (i.e., with dielectric constant 3.55) is carried out. The functionality as a defect detector is demonstrated by measuring the phase-variation in samples consisting of the uncoated *Rogers RO4003C* substrate (the reference sample) with arrays of holes of different densities.

## 1. Introduction

Many approaches for the measurement of the dielectric constant of materials and other variables related to it, e.g., material composition, or for the detection of defects in samples, using microwaves have been reported. Of particular interest are those approaches based on fully planar structures, since planar sensors are low-cost; are compatible with other technologies (e.g., microfluidics); exhibit a low profile; and can be implemented on flexible substrates, including organic substrates, such as paper, and even on recyclable or compostable materials. Additionally, the associated electronics for the generation of the microwave signals, as well as the post-processing electronic module, and, eventually, the required circuitry for wireless communication, can be integrated with the sensitive part of the sensor, typically consisting of a transmission line configuration, a set of planar resonators, or a combination of both types of elements.

Although the most typical strategy for sensing using microwaves is frequency variation [1,2,3,4,5,6,7,8,9,10,11,12,13,14,15,16,17] (the sensitive element being a resonant element), such an approach requires wideband signals for measurement (this applies also to frequency splitting sensors [18,19,20,21,22,23,24,25,26,27]), thereby increasing the cost of the associated electronics. The reason is the requirement of wideband voltage controlled oscillators (VCOs). To alleviate this limitation, single-frequency sensors constitute a good alternative. In such sensors, a single-tone harmonic signal suffices for sensing. Examples of single-frequency sensors based on coupling modulation have been reported [28,29,30,31,32,33,34,35,36,37,38,39,40,41,42]. In such sensors, the working principle is the variation of the transmission coefficient of a transmission line at the frequency of operation caused by a variation in the coupling level between such a line and a sensing resonant element (or set of resonant elements). Such sensors have been mainly devoted to the measurement of linear and angular displacements and velocities, as far as a relative displacement between the line and the resonant element modifies the coupling level between both elements.

An alternative approach for sensing using a harmonic signal is phase-variation [43,44,45,46,47,48,49,50]. Phase-variation sensors are especially suited for material characterization, including dielectric constant measurements, determination of material composition, and defect detection, among others. In these sensors, the working principle is the variation of the electrical length of the sensing line caused by the presence of the so-called material (or sample) under test (MUT) on top of it. Such change in the electrical length of the sensing line in turn modifies the phase of the transmission coefficient (transmission-mode sensors) or the phase of the reflection coefficient (reflective-mode sensors) of the whole sensing structure, which are easily measurable quantities.

Highly sensitive phase-variation sensors based on ordinary lines have been reported at the expense of a relatively large size for the sensing region [44,45] (it is well known that the sensitivity of the phase of a transmission line with the dielectric constant of the material surrounding it increases by increasing the length of the line and/or the frequency of the injected signal). As an alternative to ordinary lines, artificial lines exhibiting strong dispersion are also useful for the implementation of phase-variation sensors devoted to material characterization. Thus, composite right- and left-handed (CRLH) transmission lines [43], electro-inductive-wave (EIW) transmission lines [46], and slow-wave transmission lines [48] have been applied to the implementation of highly sensitive dielectric constant sensors. Despite the fact that with these sensors very good sensitivity with a relatively small sensing area was obtained, the design of artificial lines is not exempt of a certain complexity. Moreover, since the dispersive behavior of artificial lines is limited by the implementable values of the reactive elements loading the lines (CRLH or slow-wave lines), or by the effects of losses (EIW lines), increasing the sensitivity up to extraordinary values ultimately means to increase the length of the lines, that is, the size of the sensing region.

Recently, a different approach for the implementation of phase-variation sensors operating in reflection has been reported [49] (other reflective-mode sensors operating differentially are presented in [51]). These sensors consist of an open-ended stepped-impedance transmission line, where the sensitive region is merely the open-ended line section, which should be either a high-impedance 90° line or a low-impedance 180° line in order to optimize the sensitivity. The step-impedance discontinuities further contribute to enhancing the sensitivity, provided the line sections with alternating high and low impedance exhibit electrical lengths of 90°, as demonstrated in [49]. Indeed, there is a multiplicative effect, and the sensitivity can be enhanced as one wishes, at the expense of a degradation in the linearity. Importantly, such sensitivity enhancement does not represent an increase in the size of the sensing region, since it is achieved by merely adding further 90° stages to the stepped-impedance transmission line structure. Moreover, sensor design is extremely simple, since these phase-variation sensors are made of a cascade of ordinary lines with alternating high and low impedance. A limited aspect, as mentioned, is the linearity. For this main reason, in the design of the sensor, a reference dielectric constant for the MUT should be chosen. Consequently, the sensitivity can be optimized in the vicinity of such a reference dielectric constant value, *ε_REF_*. In [49], the reference dielectric constant was considered to be the one of air (*ε_REF_* = 1). In this paper, it is demonstrated that the sensitivity can be optimized for arbitrary values of *ε_REF_*. Therefore, not only variations in the dielectric constant of the MUT in the vicinity of the reference value can be accurately measured, but it is also possible to use the sensor as defect detector. With such later functionality, it is expected that the sensor is able to determine if the so-called reference sample is absent of defects or not (since, typically, such defects are manifested as tiny variations in the effective dielectric constant of the reference sample).

## 2. Sensor Design and Fabrication

**Figure 1:**
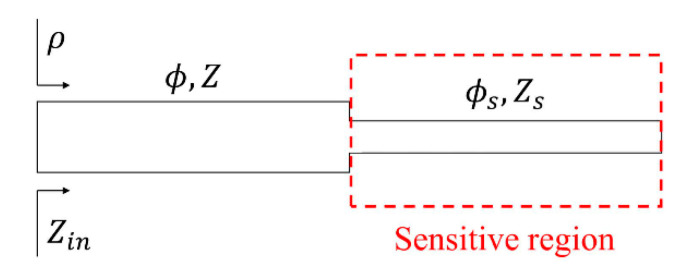
Topology/schematic of the step-impedance open-ended line reflective-mode phase-variation sensors. A single step-impedance discontinuity is considered, but further discontinuities are possible.

The structure of the considered reflective-mode phase-variation sensors is depicted in Figure 1. It consists of an open-ended sensing line with phase *ϕ_s_* and characteristic impedance *Z_s_*, cascaded to a transmission line section (the design line) with phase *ϕ* and characteristic impedance *Z*. In [49], it was demonstrated that for sensitivity optimization, the phase of the design line must be set to *ϕ* = 90°, whereas the phase of the sensing line must be set to either *ϕ_s_* = 90° or *ϕ_s_* = 180°. Moreover, the characteristic impedances of such line sections should be set to as much extreme values as possible. Specifically, if the phase of the sensing line is set to 90°, then the characteristic impedance of such a line must be high, as compared to *Z*_0_, the reference impedance of the ports, whereas the characteristic impedance of the design line must be set to a low value. Conversely, for the 180° sensing line, the impedances should satisfy *Z_s_* < *Z*_0_ and *Z* > *Z*_0_, in order to optimize the sensitivity.

As it was reported in [49], a variation in the dielectric constant of a certain MUT in contact with the sensing line modifies both the electrical length and the characteristic impedance of such a line. Thus, the sensitivity, or variation of the phase of the reflection coefficient, *ϕ_ρ_*, the output variable, with the dielectric constant of the MUT, *ε_MUT_*, the input variable, can be expressed as

(1) $$S=\frac{d\phi_{\rho }}{d\varepsilon_{MUT}}=\frac{d\phi_{\rho }}{d\phi_{s}}\cdot \frac{d\phi_{s}}{d\varepsilon_{MUT}}+\frac{d\phi_{\rho }}{dZ_{s}}\cdot \frac{dZ_{s}}{d\varepsilon_{MUT}}$$

An exhaustive analysis carried out in [49] demonstrates that for the optimum phases of the line sections of the sensor (indicated at the beginning of this section), the last term in Equation (1) is null, since *dϕ_ρ_*,/*dZ_s_* = 0 for these phases. Evaluation of the sensitivity for these specific phases gives

(2a) $$S=-\frac{Z_{0}Z_{s}\left(1-F\right)}{2Z^{2}\varepsilon_{eff}}\phi_{s}$$

for *ϕ_s_* = 90°, and

(2b) $$S=-\frac{\;Z^{2}\left(1-F\right)}{2Z_{0}Z_{s}\varepsilon_{eff}}\phi_{s}$$

for *ϕ_s_* = 180°, in both cases with *ϕ* = 90°. In Equation (2a,b), *ε_eff_* is the effective dielectric constant of the sensing line covered by the considered MUT, and *F* is a form factor. In microstrip technology, adopted in the present work, *ε_eff_* and *F* are given by [52]

(3) $$\varepsilon_{eff}=\frac{\varepsilon_{r}+\varepsilon_{MUT}}{2}+\frac{\varepsilon_{r}-\varepsilon_{MUT}}{2}F$$

and

(4a) $$F={\left(1+12\frac{h}{W_{s}}\right)}^{-1/2}$$

for *W_s_*/*h* ≥ 1, or by

(4b) $$F={\left(1+12\frac{h}{W_{s}}\right)}^{-1/2}+0.04{\left(1-\frac{W_{s}}{h}\right)}^{2}$$

for *W_s_*/*h* < 1. In Equation (3), *ε_r_* is the dielectric constant of the substrate, and it has been assumed that the MUT is semi-infinite in the vertical direction. In Equation (4a,b), *h* and *W_s_* are the substrate thickness and the width of the sensing line, respectively, and it is assumed that *t << h*, where *t* is the thickness of the metallic layer.

It is clear, in view of Equation (2a,b), that for sensitivity enhancement, the impedances should satisfy *Z_s_* > *Z*_0_ > *Z* for the 90° sensing line, and *Z_s_* < *Z*_0_ < *Z* for the 180° sensing line. It should be emphasized that for the sensing line, the required phase (90° or 180°) should be satisfied at the design frequency with the presence of the reference sample, with dielectric constant *ε_REF_* on top of it. By contrast, the 90° phase of the line section placed between the open-ended sensing line and the input port (design line) should be satisfied with air on top of it (as far as the sensing region is merely restricted to the sensing line, as depicted in Figure 1).

**Figure 2:**
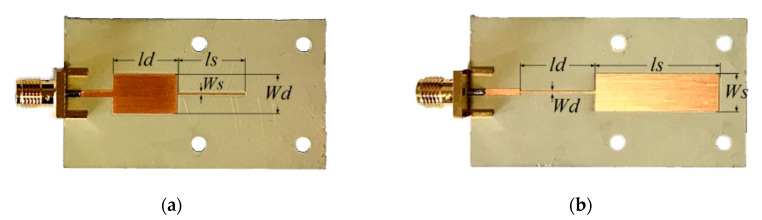
Photographs of the designed and fabricated reflective-mode phase variation sensors. (**a**) Sensor A; (**b**) sensor B. For sensor A, the dimensions (in mm) are as follows: *W_d_* = 7.5, *l_d_* = 12.8, *W_S_* = 0.235 and *l_S_* = 13.5. For sensor B, the dimensions (in mm) are as follows: *W_d_* = 0.32, *l_d_* = 14.6, W_S_ = 7.5 and *l_S_* = 24.4.

With the design guidelines for sensitivity optimization, two different sensors were designed and fabricated. In one sensor, designated as sensor A, the structure consists in a *Z_s_* = 85 Ω open-ended sensing line with phase *ϕ_s_* = 90° when the uncoated *Rogers RO4003C* substrate with *ε_REF_* = 3.55 is on top of such a line, cascaded to a *ϕ* = 90° design line with *Z* = 15 Ω characteristic impedance. The frequency of operation was set to *f*_0_ = 2 GHz, and the considered substrate for sensor implementation was the *Rogers RO3010* substrate with dielectric constant *ε_r_* = 10.2, thickness *h* = 1.27 mm and loss tangent tan δ = 0.0023. For sensor B, the substrate and operation frequency are the same, whereas the line variables are *Z_s_* = 15 Ω and *ϕ_s_* = 180° (when the same uncoated *Rogers RO4003C* substrate with *ε_REF_* = 3.55 is on top of the open-ended sensing line), and *ϕ* = 90°, *Z* = 85 Ω for the cascaded design line. With these parameters for sensors A and B, the two cases that optimize the sensitivity (high-impedance 90° and low-impedance 180° sensing lines) are considered. The photographs of both sensors, fabricated by means of an *LPKF H100* drilling machine, are depicted in Figure 2, where the relevant dimensions are indicated (see caption).

Although the main interest in the present paper is the sensitivity analysis and optimization, providing a mathematical sensing model linking the phase variation to the dielectric constant of the MUT is also insightful. Obviously, Figures 4a and 5a of next section can be used to infer the dielectric constant of the MUT from the measured phase. Mathematically, the dependence of the phase of the reflection coefficient, the output variable, with the dielectric constant of the MUT, *ε_MUT_*, is given by:

(5) $$\phi_{\rho }=2\arctan\left(\frac{Z(Z_{s}\cot\phi_{s}-Z\tan\phi )}{Z_{0}(Z+Z_{s}\tan\phi \cot\phi_{s})}\right)$$

where the dependence with *ε_MUT_* is through the sensing line parameters, *Z_s_* and *ϕ_s_*. Indeed, for the value of *ϕ* that optimizes the sensitivity (90°), Equation (5) simplifies to

(6) $$\phi_{\rho }=2\arctan\left(-\frac{Z^{2}}{Z_{0}Z_{s}}\tan\phi_{s}\right)$$

Both *ϕ_s_* and *Z_s_* depend on the effective dielectric constant of the sensing line according to

(7) $$\phi_{s}=\frac{\omega_{0}l_{s}}{c}\sqrt{\varepsilon_{eff}}$$

and

(8) $$Z_{s}=\frac{Z\prime }{\sqrt{\varepsilon_{eff}}}$$

respectively, where *Z*’ is the characteristic impedance of an hypothetical line with identical dimensions to the sensing line, but surrounded by air and with a substrate with dielectric constant *ε_r_* = 1. In Equation (7), *ω*_0_ is the angular operating frequency, and *c* is the speed of light in vacuum. The dependence of Z’ with the line geometry can be found in several textbooks [52]. On the other hand, the effective dielectric constant depends on *ε_MUT_* according to Equation (3). Thus, with the previous expressions, a mathematical model linking the input and output variable is provided.

## 3. Results

**Figure 3:**
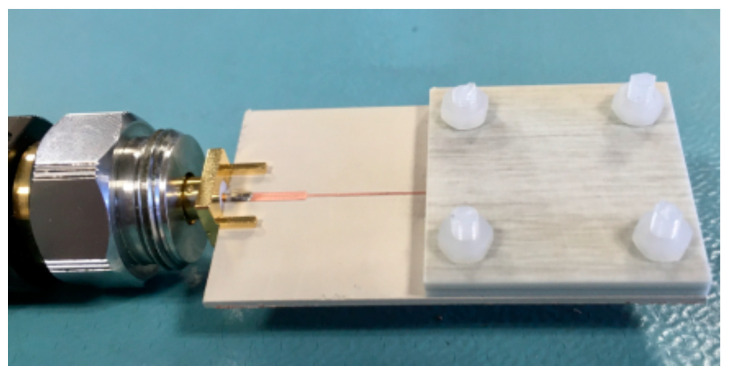
Detail of one of the sensors covered by the material under test (MUT). In order to avoid the presence of an air gap between the MUT sample and the sensing line, the sample was pressured against the sensing line by means of Teflon screws.

For both designed and fabricated sensors, we obtained the phase variation in the reflection coefficient as a function of the dielectric constant of the MUT, taking as reference the phase of the reflection coefficient when the sensing line is covered by the reference substrate (indicated before). This has been done by means of electromagnetic simulation, using the *Keysight ADS* commercial software, theoretically, using Equations (6)–(8), and experimentally. In this latter case, several MUT samples with different dielectric constant were located on top of the sensing line, as Figure 3 illustrates. The thickness of the MUTs is roughly 3 mm (achieved by stacking two samples), sufficient to consider the substrate semi-infinite in the vertical direction. By these means, the experimental conditions are identical to those of the simulations (where the MUT is considered to be vertically semi-infinite, a necessary condition for the validity of Equation (2a,b), as discussed in [49]). The phase of the reflection coefficient was obtained by means of the *Keysight 85072A* vector network analyzer. It should also be mentioned that since the *Keysight ADS* software does not permit the simulation of structures with dielectric layers of different transverse dimensions, we first simulated solely the sensing line covered with the MUT. Then, we simulated the design line alone, and, finally, we imported the results to the *Keysight ADS* schematic simulator in order to obtain the phase response of the whole structure.

**Figure 4:**
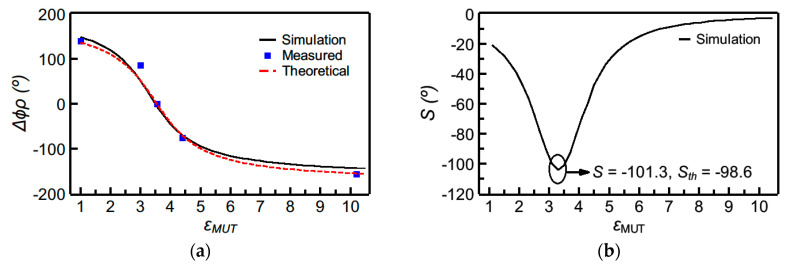
Dependence of the differential phase of the reflection coefficient (i.e., ∆*ϕ_ρ_* = *ϕ_ρ_* − *ϕ_ρ_*_,*REF*_) with the dielectric constant of the MUT (**a**) and sensitivity (**b**) for sensor A. The measured data points were obtained by covering the sensing line with the following uncoated materials: *Rogers RO3010* (*ε_MUT_* = 10.2), *FR4* (*ε_MUT_* = 4.4), *Rogers RO4003C* (*ε_MUT_* = *ε_REF_* = 3.55), *PLA* (*ε_MUT_* = 3.0, and fabricated by means of a 3D printer). Additionally, the phase of the reflection coefficient was experimentally obtained with the sensing line uncovered (*ε_MUT_*_, air_ = 1).

**Figure 5:**
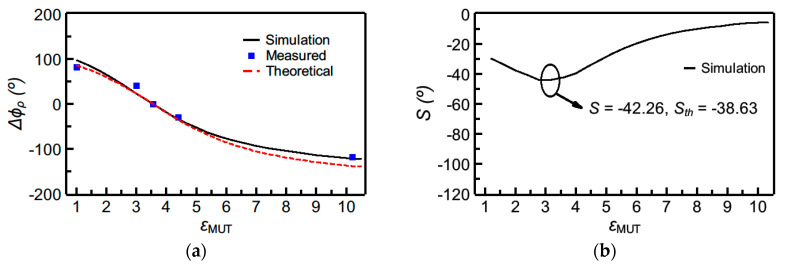
Dependence of the differential phase of the reflection coefficient (i.e., ∆*ϕ_ρ_* = *ϕ_ρ_* − *ϕ_ρ_*_,*REF*_) with the dielectric constant of the MUT (**a**) and sensitivity (**b**) for sensor B. The MUTs are identical to those of Figure 4.

Figure 4 depicts the phase variation as a function of the dielectric constant of the MUT, as well as the sensitivity (inferred from the simulated data points), for sensor A, whereas the corresponding values for sensor B are shown in Figure 5. The theoretical sensitivities, given by Equation (2a,b), evaluated at the dielectric constant of the reference MUT, are also given in the figures (and designated as *S_th_*). As can be appreciated, the agreement between the sensitivity inferred from the simulated data points and the one given by the theoretical expressions is good. The sensitivity is maximized for *ε_MUT_* being equal to the dielectric constant of the REF sample, and, consequently, these sensors are especially suited for the measurement of tiny variations of the dielectric constant in the vicinity of 3.55, the dielectric constant of the REF sample. A very high value of the maximum sensitivity (*S* = −101.3°) in the simulated sensitivity for sensor A was obtained, by virtue of the stepped-impedance configuration.

**Figure 6:**
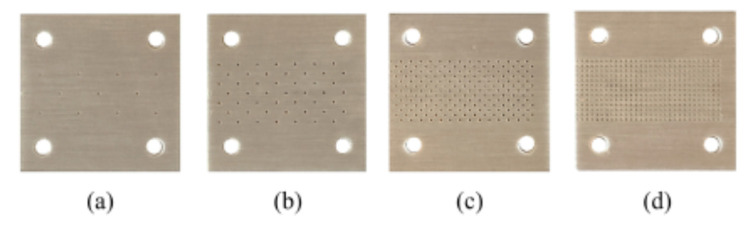
Reference sample with arrays of holes of different densities drilled across the substrate. From sample (**a**–**d**), the density of holes increases.

Functionality as a defect detector of the proposed sensing structure relies on the variation of the effective dielectric constant of the sample under test, caused by the presence of defects or anomalies. In order to demonstrate the potential of the designed sensor to detect defects in samples, we generated defects in the reference sample, the uncoated slab of *Rogers RO4003C* substrate, consisting of arrays of holes across the samples, of different densities (Figure 6).

**Figure 7:**
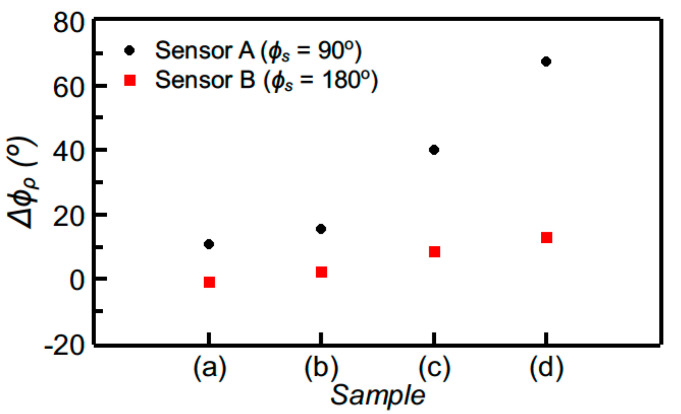
Differential phase of the reflection coefficient measured by covering the sensing line of sensors A and B with the samples of Figure 6.

**Figure 8:**
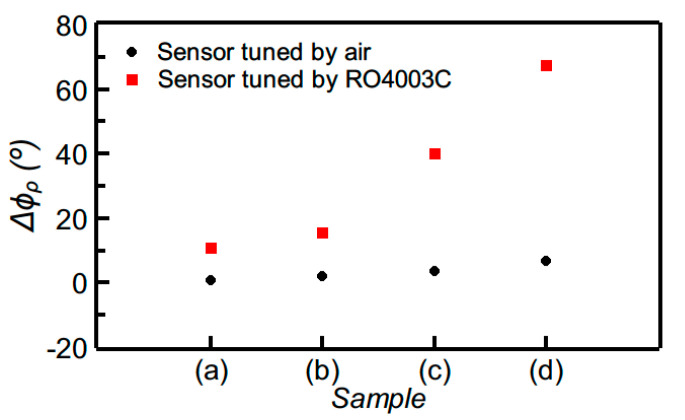
Comparison of the measured differential phase of the reflection coefficient for sensor A and for the equivalent sensor, but with the sensing line exhibiting a phase of 90° when it is uncovered (i.e., tuned by air).

The measured phase of the reflection coefficient for both sensors A and B corresponding to the different “defected” samples is depicted in Figure 7. The sensitivity is superior in sensor A, which demonstrates its capability of perfectly detecting the presence of the sparser array of holes in the sample (a) of Figure 6a, as revealed by the significant variation experienced by the phase of the reflection coefficient for that sample. Obviously, this high sensitivity in detecting tiny defects is due to the fact that the sensors were designed by considering the “un-defected” sample as reference material, i.e., with the optimum phases of the sensing line (90° or 180° for sensor A or B, respectively) when such a line is covered with such material. In contrast, if the sensing line is designed with 90° or 180° phase when it is covered by air, the capability to detect small changes (“defects”) in samples with dielectric constant substantially different to the one of air, decreases drastically. This is visible in Figure 8, where the comparison of the response of sensor A with that of the equivalent sensor, but tuned by air, is depicted. Thus, in summary, these results demonstrate that detecting the presence of defects or abnormalities in samples, in comparison to a well-known reference, is possible by properly designing the sensor (i.e., by taking into account the dielectric constant of the reference sample, and by designing the sensing line with the required phase, either 90° or 180°, for sensitivity optimization).

It should be mentioned that, alternatively, the reported sensors could have been implemented in coplanar waveguide technology (CPW). In [50], it was demonstrated that the intrinsic sensitivity of CPWs is larger than that of microstrip lines. The reason is that the electric field generated by a CPW has a strong presence on top of the line, as compared to microstrip lines. However, microstrip line technology offers backside isolation. Moreover, sensitivity enhancement can be improved, if required, regardless of the specific technology, by adding further cascaded high/low impedance 90° line sections.

Let us mention, to finish the present section, that the phase, like the frequency, is robust against the effects of electromagnetic interference and noise, at least as compared to the magnitude (e.g., the magnitude of a transmission or a reflection coefficient, a typical output variable in microwave sensors). In this regard, phase-variation sensors, as those reported in this paper, or frequency-variation sensors, are more tolerant than coupling-modulation sensors (or other sensors using either the magnitude of the reflection or transmission coefficient) to electromagnetic interferences and noise. Concerning the effects of cross sensitivities caused, e.g., by changes in temperature or humidity, phase-variation and frequency-variation sensors are not as good as differential-mode sensors, or sensors based on symmetry properties. This is because changes in environmental factors occur at scales much larger than the typical size of sensors, and, therefore, such changes are seen as common-mode stimulus by differential-mode sensors or by sensors based on symmetry properties. Nevertheless, the proposed phase-variation sensors exhibit relevant advantages, such as operation at a single frequency, robustness against electromagnetic interference and noise, one-port device operation (in reflection mode), and, most importantly, highly achievable sensitivities.

## 4. Conclusions

In conclusion, highly sensitive reflective-mode phase-variation sensors devoted to the accurate measurement of the dielectric constant of MUT samples in the vicinity of a certain predetermined value were reported in this paper. It has also been demonstrated that, thanks to such high sensitivity, the sensors are also useful as defect detectors or comparators, able to discern if a certain sample is identical or not to a reference sample. Due to the fact that the sensors merely consist of an open-ended sensing line (with either high characteristic impedance and 90° phase, or low impedance and 180° phase), cascaded to a 90° design line with high impedance contrast in regard to the sensing line, sensor design and fabrication are very simple. Two prototype sensors were designed, fabricated, and applied to the dielectric characterization of different MUT samples, as well as to the detection of defects (consisting of drilled holes) in a reference sample. In one of the sensors, the maximum sensitivity was found to be as high as −101.3°, but this value can be further enhanced by cascading further 90° stages (with as much impedance contrast as possible) to the designed and fabricated structure. By redesigning the sensor and adding a fluidic channel on top of the sensing line, the application of the structure to the characterization of liquid samples, particularly to the determination of small variations of solute content in liquid solutions, is envisaged. In particular, these sensors may find application in industrial processes, such as wine fermentation and medical diagnosis and monitoring, e.g., the determination of variations of electrolyte content in urine or blood.

## References

[1] Mandel C., Kubina B., Schüßler M., Jakoby R. Passive chipless wireless sensor for two-dimensional displacement measurement. Proceedings of the 41st European Microwave Conference.

[2] Puentes M. (2014). Planar Metamaterial Based Microwave Sensor Arrays for Biomedical Analysis and Treatment.

[3] Ebrahimi A., Withayachumnankul W., Al-Sarawi S., Abbott D. (2013). High-sensitivity metamaterial-inspired sensor for microfluidic dielectric characterization. IEEE Sens. J..

[4] Schüßler M., Mandel C., Puentes M., Jakoby R. (2012). Metamaterial inspired microwave sensors. IEEE Microw. Mag..

[5] Boybay M.S., Ramahi O.M. (2012). Material characterization using complementary split-ring resonators. IEEE Trans. Instrum. Meas..

[6] Lee C.S., Yang C.L. (2014). Complementary split-ring resonators for measuring dielectric constants and loss tangents. IEEE Microw. Wirel. Compon. Lett..

[7] Yang C.L., Lee C.S., Chen K.W., Chen K.Z. (2015). Noncontact measurement of complex permittivity and thickness by using planar resonators. IEEE Trans. Microw. Theory Tech..

[8] Withayachumnankul W., Jaruwongrungsee K., Tuantranont A., Fumeaux C., Abbott D. (2013). Metamaterial-based microfluidic sensor for dielectric characterization. Sens. Actuators A Phys..

[9] Salim A., Lim S. (2016). Complementary split-ring resonator-loaded microfluidic ethanol chemical sensor. Sensors.

[10] Su L., Mata-Contreras J., Vélez P., Fernández-Prieto A., Martín F. (2018). Analytical method to estimate the complex permittivity of oil samples. Sensors.

[11] Abdolrazzaghi M., Zarifi M.H., Daneshmand M. Sensitivity Enhancement of Split Ring Resonator Based Liquid Sensors. Proceedings of the 2016 IEEE SENSORS.

[12] Abdolrazzaghi M., Zarifi M.H., Pedrycz W., Daneshmand M. (2016). Robust ultra-high resolution microwave planar sensor using fuzzy neural network approach. IEEE Sens. J..

[13] Zarifi M.H., Daneshmand M. (2017). Monitoring solid particle deposition in lossy medium using planar resonator sensor. IEEE Sens. J..

[14] Zarifi M.H., Deif S., Abdolrazzaghi M., Chen B., Ramsawak D., Amyotte M., Vahabisani N., Hashisho Z., Chen W., Daneshmand M. (2017). A microwave ring resonator sensor for early detection of breaches in pipeline coatings. IEEE Trans. Ind. Electron..

[15] Abdolrazzaghi M., Daneshmand M., Iyer A.K. (2018). Strongly enhanced sensitivity in planar microwave sensors based on metamaterial coupling. IEEE Trans. Microw. Theory Tech..

[16] Zarifi M.H., Sadabadi H., Hejazi S.H., Daneshmand M., Sanati-Nezhad A. (2018). Noncontact and nonintrusive microwave-microfluidic flow sensor for energy and biomedical engineering. Sci. Rep..

[17] Ebrahimi A., Scott J., Ghorbani K. (2019). Ultrahigh-Sensitivity Microwave Sensor for Microfluidic Complex Permittivity Measurement. IEEE Trans. Microw. Theory Tech..

[18] Horestani A.K., Naqui J., Shaterian Z., Abbott D., Fumeaux C., Martín F. (2014). Two-dimensional alignment and displacement sensor based on movable broadside-coupled split ring resonators. Sens. Actuators A Phys..

[19] Naqui J., Damm C., Wiens A., Jakoby R., Su L., Martín F. Transmission lines loaded with pairs of magnetically coupled stepped impedance resonators (SIRs): Modeling and application to microwave sensors. Proceedings of the IEEE MTT-S Int. Microwave Symposium.

[20] Su L., Naqui J., Mata-Contreras J., Martín F. (2014). Modeling metamaterial transmission lines loaded with pairs of coupled split-ring resonators. IEEE Antennas Wirel. Propag. Lett..

[21] Su L., Naqui J., Mata J., Martín F. Dual-band epsilon-negative (ENG) transmission line metamaterials based on microstrip lines loaded with pairs of coupled complementary split ring resonators (CSRRs): Modeling, analysis and applications. Proceedings of the 2015 9th International Congress on Advanced Electromagnetic Materials in Microwaves and Optics (METAMATERIALS).

[22] Su L., Naqui J., Mata-Contreras J., Vélez P., Martín F. Transmission line metamaterials based on pairs of coupled split ring resonators (SRRs) and complementary split ring resonators (CSRR): A comparison to the light of the lumped element equivalent circuits. Proceedings of the International Conference on Electromagnetics for Advanced Applications (ICEAA 2015).

[23] Su L., Naqui J., Mata-Contreras J., Martín F. (2015). Modeling and applications of metamaterial transmission lines loaded with pairs of coupled complementary split-ring resonators (CSRRs). IEEE Antennas Wirel. Propag. Lett..

[24] Naqui J., Damm C., Wiens A., Jakoby R., Su L., Mata-Contreras J., Martín F. (2016). Transmission lines loaded with pairs of stepped impedance resonators: Modeling and application to differential permittivity measurements. IEEE Trans. Microw. Theory Tech..

[25] Su L., Mata-Contreras J., Vélez P., Martín F. (2016). Splitter/combiner microstrip sections loaded with pairs of complementary split ring resonators (CSRRs): Modeling and optimization for differential sensing applications. IEEE Trans. Microw. Theory Tech..

[26] Vélez P., Su L., Grenier K., Mata-Contreras J., Dubuc D., Martín F. (2017). Microwave microfluidic sensor based on a microstrip splitter/combiner configuration and split ring resonators (SRRs) for dielectric characterization of liquids. IEEE Sens. J..

[27] Ebrahimi A., Scott J., Ghorbani K. (2018). Differential sensors using microstrip lines loaded with two split-ring resonators. IEEE Sens. J..

[28] Naqui J., Durán-Sindreu M., Martín F. (2011). Novel sensors based on the symmetry properties of split ring resonators (SRRs). Sensors.

[29] Naqui J., Durán-Sindreu M., Martín F. On the symmetry properties of coplanar waveguides loaded with symmetric resonators: Analysis and potential applications. Proceedings of the 2012 IEEE/MTT-S International Microwave Symposium Digest.

[30] Naqui J., Durán-Sindreu M., Martín F. (2012). Alignment and position sensors based on split ring resonators. Sensors.

[31] Naqui J., Durán-Sindreu M., Martín F. Transmission lines loaded with bisymmetric resonators and applications. Proceedings of the IEEE MTT-S International Microwave Symposium Digest.

[32] Horestani A.K., Fumeaux C., Al-Sarawi S.F., Abbott D. (2012). Displacement sensor based on diamond-shaped tapered split ring resonator. IEEE Sens. J..

[33] Horestani A.K., Abbott D., Fumeaux C. (2013). Rotation sensor based on horn-shaped split ring resonator. IEEE Sens. J..

[34] Naqui J., Martı F. (2013). Transmission lines loaded with bisymmetric resonators and their application to angular displacement and velocity sensors. IEEE Trans. Microw. Theory Tech..

[35] Ebrahimi A., Withayachumnankul W., Al-Sarawi S.F., Abbott D. (2014). Metamaterial-inspired rotation sensor with wide dynamic range. IEEE Sens. J..

[36] Naqui N., Durán-Sindreu M., Martín F. Transmission lines loaded with folded stepped impedance resonators (SIRs): modelling and applications. Proceedings of the Sixth International Congress on Advanced Electromagnetic Materials in Microwaves and Optics (Metamaterials 2012).

[37] Horestani A.K., Naqui J., Abbott D., Fumeaux C., Martín F. (2014). Two-dimensional displacement and alignment sensor based on reflection coefficients of open microstrip lines loaded with split ring resonators. Electron. Lett..

[38] Naqui J., Martín F. (2013). Angular displacement and velocity sensors based on electric-LC (ELC) loaded microstrip lines. IEEE Sens. J..

[39] Naqui J., Coromina J., Karami-Horestani A., Fumeaux C., Martín F. (2015). Angular displacement and velocity sensors based on coplanar waveguides (CPWs) loaded with S-shaped split ring resonators (S-SRR). Sensors.

[40] Naqui J., Martín F. Application of broadside-coupled split ring resonator (BC-SRR) loaded transmission lines to the design of rotary encoders for space applications. Proceedings of the IEEE MTT-S International Microwave Symposium.

[41] Mata-Contreras J., Herrojo C., Martín F. (2017). Application of split ring resonator (SRR) loaded transmission lines to the design of angular displacement and velocity sensors for space applications. IEEE Trans. Microw. Theory Tech..

[42] Mata-Contreras J., Herrojo C., Martín F. (2018). Detecting the rotation direction in contactless angular velocity sensors implemented with rotors loaded with multiple chains of resonators. IEEE Sens. J..

[43] Damm C., Schüßler M., Puentes M., Maune H., Maasch M., Jakoby R. Artificial transmission lines for high sensitive microwave sensors. Proceedings of the IEEE Sensors.

[44] Ferrández-Pastor F., García-Chamizo J., Nieto-Hidalgo M. (2017). Electromagnetic differential measuring method: Application in microstrip sensors developing. Sensors.

[45] Muñoz-Enano J., Vélez P., Gil M., Martín F. (2019). An Analytical Method to Implement High Sensitivity Transmission Line Differential Sensors for Dielectric Constant Measurements. IEEE Sens. J..

[46] Gil M., Vélez P., Aznar-Ballesta F., Muñoz-Enano J., Martín F. (2020). Differential Sensor based on Electro-Inductive Wave (EIW) Transmission Lines for Dielectric Constant Measurements and Defect Detection. IEEE Trans. Antennas Propag..

[47] Muñoz-Enano J., Vélez P., Gil M., Mata-Contreras J., Martín F. (2020). Differential-mode to common-mode conversion detector based on rat-race couplers: Analysis and application to microwave sensors and comparators. IEEE Trans. Microw. Theory Tech..

[48] Coromina J., Muñoz-Enano J., Vélez P., Ebrahimi A., Scott J., Ghorbani K., Martín F. Capacitively-Loaded Slow-Wave Transmission Lines for Sensitivity Improvement in Phase-Variation Permittivity Sensors. Proceedings of the 50th European Microwave Conference.

[49] Muñoz-Enano J., Vélez P., Gil M., Martín F. (2020). On the sensitivity of reflective-mode phase variation sensors based on open-ended stepped-impedance transmission lines: Theoretical analysis and experimental validation. IEEE Trans. Microw. Theory Tech..

[50] Su L., Muñoz-Enano J., Vélez P., Casacuberta P., Gil M., Martín F. (2020). Highly sensitive phase variation sensors based on step-impedance coplanar waveguide (CPW) transmission lines for dielectric characterization. IEEE Sens. J..

[51] Ebrahimi A., Tovar-Lopez F., Scott J., Ghorbani K. (2020). Differential microwave sensor for characterization of glycerole-water solutions. Sens. Actuators B Chem..

[52] Pozar D.M. (2011). Microwave Engineering.

